# IbpB‐bound substrate release in living cells as revealed by unnatural amino acid‐mediated photo‐crosslinking

**DOI:** 10.1002/2211-5463.12957

**Published:** 2020-09-01

**Authors:** Xiaodong Shi, Anastasia N. Ezemaduka

**Affiliations:** ^1^ Jiangsu Province Key Laboratory of Anesthesiology and Jiangsu Province Key Laboratory of Anesthesia and Analgesia Application Xuzhou Medical University China; ^2^ Key Laboratory of Wetland Ecology and Environment Northeast Institute of Geography and Agroecology Chinese Academy of Sciences Changchun China

**Keywords:** IbpB, photo‐crosslinking, small heat shock proteins, substrate binding, unnatural amino acid

## Abstract

Small heat shock proteins (sHSPs) are known to bind non‐native substrates and prevent irreversible aggregation in an ATP‐independent manner. However, the dynamic interaction between sHSPs and their substrates *in vivo* is less studied. Here, by utilizing a genetically incorporated crosslinker, we characterized the interaction between sHSP IbpB and its endogenous substrates in living cells. Through photo‐crosslinking analysis of five Bpa variants of IbpB, we found that the substrate binding of IbpB in living cells is reversible upon short‐time exposure at 50 °C. Our data provide *in vivo* evidence that IbpB engages in dynamic substrate release under nonstress conditions and suggest that photo‐crosslinking may be a suitable method for investigating dynamic interaction between molecular chaperones and their substrates in living cells.

## Graphical abstract

Small heat shock proteins (sHSPs) are known to bind partially folded proteins under stress conditions. In this study, by using photo‐crosslinking, we showed that the substrate binding of sHSP IbpB in living cells is reversible upon short‐time exposure at elevated temperature, providing *in vivo* evidence on the existence of substrate protein release at nonstress conditions.

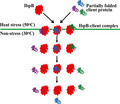

**Abbreviations**
Bpa: *p*‐benzoyl‐l‐phenylalanine
*E. coli*: *Escherichia coli*
sHSPs: small heat shock proteins

Molecular chaperone proteins play key roles in maintaining proteostasis *in vivo* by assisting the folding of substrate proteins, preventing or reversing misfolding proteins, and the degradation of their misfolded forms [1, 2]. Small heat shock proteins (sHSPs), as a conserved family of molecular chaperone with a low molecular mass of 12–43 kDa, are present in all forms of life [3]. The ability of sHSPs to confer resistance on cells under various stress conditions has been widely reported [4, 5, 6, 7]. They are in proteostasis network referred to as first line stress defenders, binding non‐native substrate proteins and holding them in a folding‐competent state as ‘holdases’, which might be subsequently refolded with the assistance of other ATP‐dependent molecular chaperones such as Hsp60, Hsp70, and Hsp100 [3, 8, 9, 10, 11, 12, 13]. It has been shown that the sHSPs associate with protein aggregates, altering their biochemical properties, and subsequently facilitate efficient disaggregation and refolding [9, 14, 15, 16, 17]. However, the basic understandings about the interaction between sHSPs and their substrate proteins have been obtained mostly from *in vitro* studies [3], raising the question of their unique *in vivo* characteristics. Such unresolved scientific questions regarding the unique properties as regard to mode of operation of sHSPs *in vivo* include among others, what kind of dynamic changes take place in the interaction between sHSPs and their endogenous substrate proteins and, whether such interaction is reversible or irreversible? The answers to these questions may further our understanding of the molecular mechanisms for sHSPs to function *in vivo*.

To address these questions, we chose the inclusion body binding protein IbpB, a sHSP from *Escherichia coli* (*E. coli*) as a model to investigate the interaction characteristics of sHSPs and their substrate proteins in living cells. IbpB, which was initially identified as a component of inclusion bodies [18, 19] and later reported to be present in heat shock formed aggregates [20], confers *E. coli* cells resistance against stresses [21, 22]. In our previous studies, substrate‐binding residues of IbpB were characterized by using *in vivo* site‐specific photo‐crosslinking as mediated by the genetically incorporated unnatural amino acid *p*‐benzoyl‐l‐phenylalanine (Bpa) [23], and the substrate proteins captured by Bpa were identified to have remarkable preference for translation‐related proteins and metabolic enzymes [24]. In the present study, we chose a total of five IbpB Bpa variants to investigate the interaction features of IbpB‐substrate in *E. coli* cells subjected to both stress and nonstress conditions by adopting *in vivo* site‐specific photo‐crosslinking, as it is capable of covalently capturing transiently or weakly interacting proteins [25]. Our results revealed that the IbpB‐substrate interaction in living cells is reversible upon short‐time exposure to 50 °C, providing the *in vivo* evidence on the existence of substrate protein release at nonstress conditions.

## Materials and methods

### Bacterial strains, plasmid construction, and protein expression

*Escherichia coli* BW25113‐Δ*ibpB* strain was obtained from Nara Institute of Science and Technology in Japan. *E. coli* DH5α cells were used for gene manipulation. The recombinant plasmids, expressing wild‐type or Bpa variants of IbpB with a tag of six histidine residues being added at the C terminus, were constructed as described previously [23]. The pSup‐BpaRS‐6TRN plasmid, expressing the orthogonal aminoacyl‐tRNA synthetase/tRNA pair for the incorporation of Bpa into IbpB, was cotransformed with the recombinant plasmid into *E. coli* Δ*ibpB* cells. Cells were cultured at 30 °C in the presence of appropriate antibiotics (final concentrations of 100 μg·mL^−1^ ampicillin, 50 μg·mL^−1^ kanamycin, and 50 μg·mL^−1^ chloramphenicol; Sigma, St Louis, MO, USA), 1 mm Bpa (Bachem AG, Bubendorf, Switzerland), and 0.02% arabinose to induce protein expression.

### Chaperone‐like activity assay for Bpa variants of IbpB

The chaperone‐like activity of each Bpa variant was measured to determine its capacity to suppress the heat‐induced aggregation of whole cell extract of *E. coli ΔibpB* cells. Briefly, the *E. coli ΔibpB* cells overexpressing each Bpa variant of IbpB‐His_6_ were cultured overnight at 30 °C. Cells were harvested by centrifugation, washed twice, and resuspended in 20 mm Tris/HCl buffer (pH 8.0), lysed by sonication, and centrifuged at 13 000 ***g*** for 30 min at 4 °C to remove the cell debris. The resultant whole cell extract was incubated at 50 °C for 1 h. After heat shock treatment, 400 μL cell extract was divided into two parts and 20 μL was taken from one part as the whole proteins. The other part was centrifuged at 13 000 ***g*** for 30 min at 4 °C; then, 20 μL was taken from the supernatant as the soluble proteins fraction. After discarding all the supernatants, the pellet was washed twice and resuspended using 200 μL Tris/HCl; then, 20 μL was taken as the insoluble proteins fraction. The whole cell extract, soluble proteins, and insoluble protein aggregates were subjected to 10% Tricine/SDS/PAGE and Coomassie blue staining analysis. The relative chaperone‐like activity was defined by the percentage of the soluble protein of the whole cell extract according to the semiquantification results based on the corresponding Coomassie blue staining results. The mean gel density analysis was measured using imagej software [26]

### Bpa‐mediated *in vivo* photo‐crosslinking

The *E. coli* Δ*ibpB* cells transformed with pBAD carrying the gene of IbpB Bpa variant and pSup‐BpaRS‐6TRN plasmid were initially grown at 30 °C to *A*_600_ = 0.4; induced by arabinose for 2 h to express the IbpB variant protein; washed twice using fresh LB to remove arabinose; incubated at 50 °C for 10 min; and transferred back to 30 °C for a prolonged incubation. Cultures were taken out at indicated time points and immediately transferred to 24‐well plate before being subjected to UV irradiation at 365 nm for 10 min using a Hoefer UVC 500 crosslinker. The cells were lysed, analyzed by 10% Tricine/SDS/PAGE, and then immunoblotted with anti‐His tag monoclonal antibody.

### Semiquantification of relative substrate binding

The relative levels of photo‐crosslinked substrates of each Bpa variant were calculated as the percentage of IbpB crosslinked to substrates based on the immunoblotting results. It should be mentioned that the portion of monomeric, dimeric, and trimeric forms of IbpB Bpa variant was subtracted from the total crosslinked protein products during image processing using imagej software.

## Results

### Incorporation of the unnatural amino acid Bpa into the N‐terminal arm of IbpB at five selected individual position

**Fig. 1:**
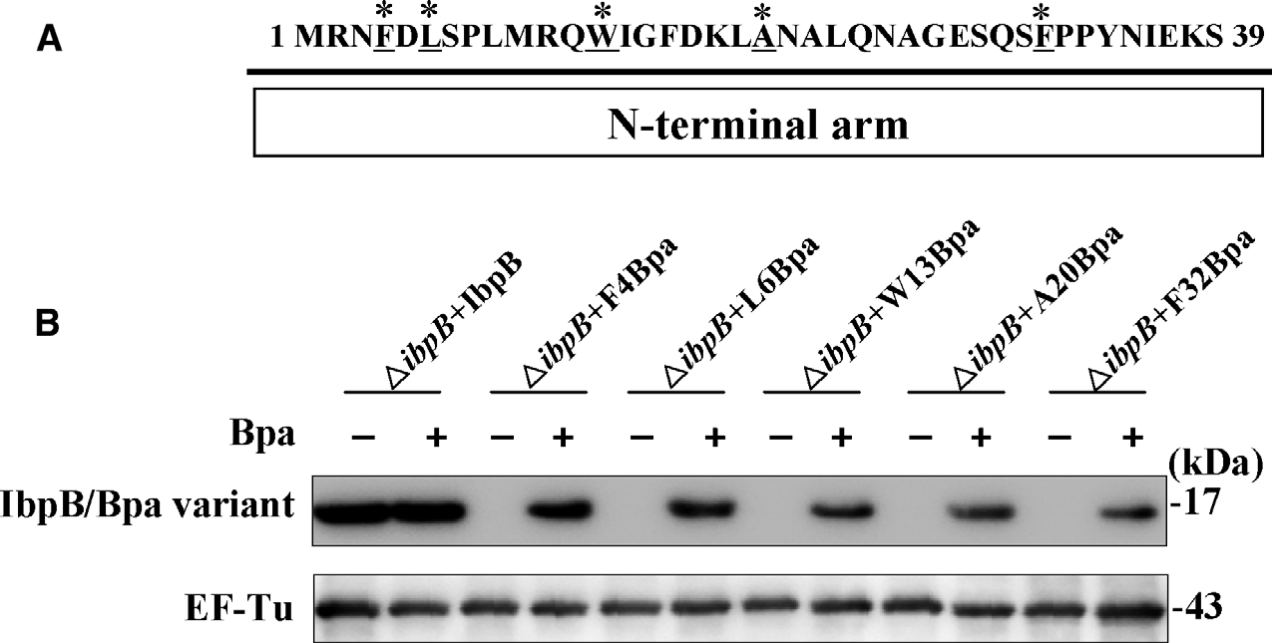
Incorporation of Bpa into the N‐terminal arm of IbpB at five selected individual position. (A) The five selected residue positions at the N‐terminal arm (amino acid 1–39) were chosen to incorporate Bpa as indicated by asterisks and underlines. (B) Determination of the expression of five IbpB Bpa variants in *Escherichia coli* BW25113‐Δ*ibpB* strain. The IbpB/Bpa variant was induced by arabinose in the absence/presence of Bpa. IbpB/Bpa variant expression was immunoblotted with anti‐His tag monoclonal antibody. The internal control EF‐Tu was immunoblotted with anti‐EF‐Tu monoclonal antibody.

The interaction between IbpB and its substrate proteins in *E. coli* cells at 30 °C and 50 °C has been investigated by utilizing genetically incorporated photo‐crosslinker Bpa [23]. Given that the substrate protein binding of IbpB was shown to be enhanced upon temperature elevation, it is of interest to determine whether a decrease in growth temperature will reversely weaken such interactions. To address this point, five individual residue positions (Phe‐4, Leu‐6, Trp‐13, Ala‐20, and Phe‐32 as shown in Fig. 1A) from the N‐terminal arm of IbpB were selected for Bpa incorporation because these IbpB Bpa variants have been shown to participate primarily in homo‐oligomerization at 30 ^o^C and switch to substrate binding at 50 °C [23], making it easy to compare the changes in substrate binding of IbpB when temperature rises from 30 °C to 50 °C and then falls back to 30 °C. To avoid the interference of endogenous IbpB, *E. coli* BW25113‐Δ*ibpB* strain was used to express the Bpa variants. Besides, these Bpa variants were all expressed with a tag of six histidine residues being added at the C terminus for the convenience of western immunoblot analysis. As displayed in Fig. 1B, the expression of five IbpB variants cannot be detected unless by the addition of Bpa, while the expression of wild‐type IbpB is independent of Bpa, confirming the successful incorporation of unnatural amino acid Bpa into the selected position.

### The chaperone‐like activity of IbpB is barely affected by incorporation of Bpa at five selected individual residue positions

**Fig. 2:**
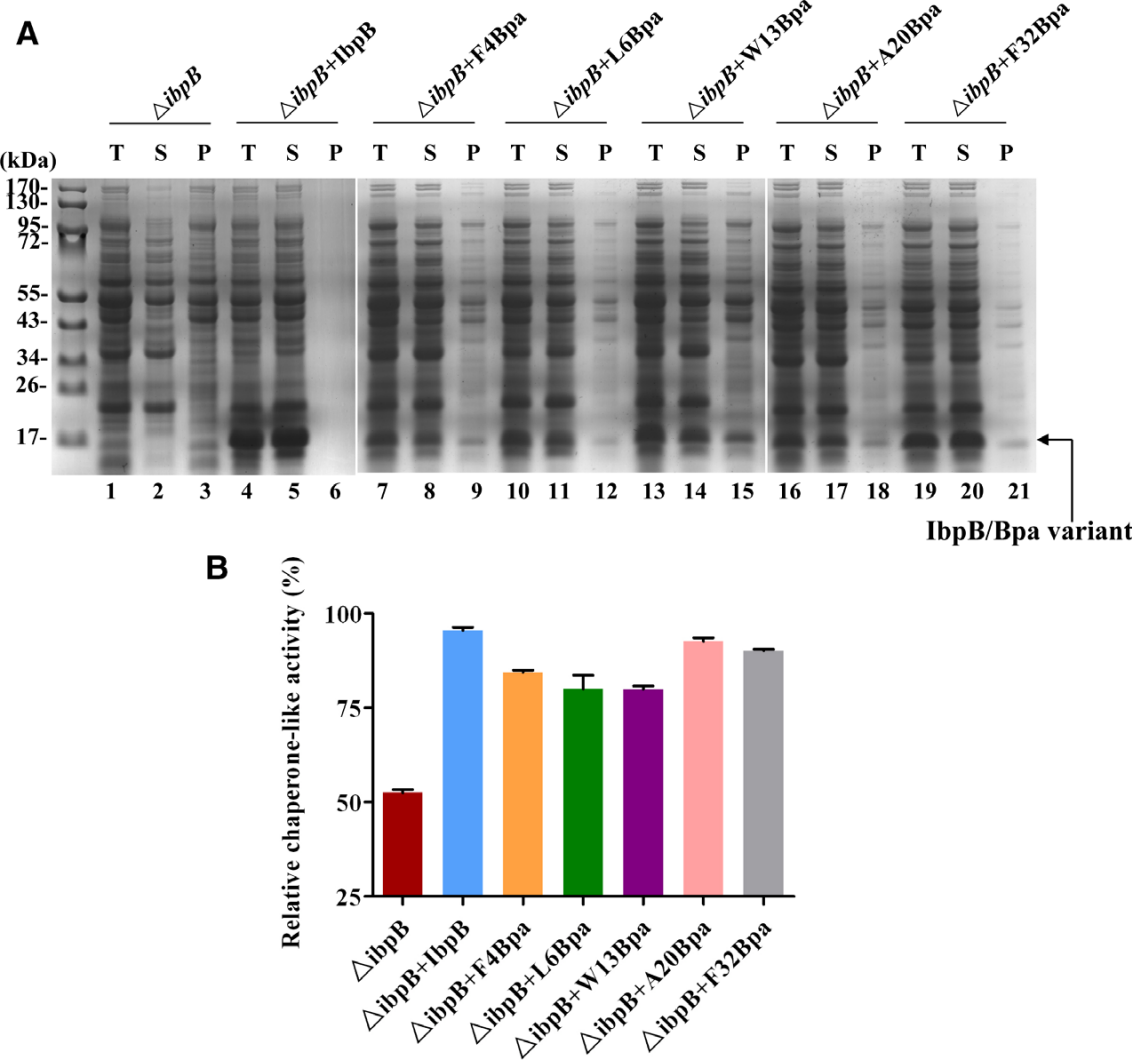
Determination of the chaperone‐like activities of IbpB Bpa variants. (A) The whole cell extract (T) of Δ*ibpB* cells expressing his‐tagged wild‐type or Bpa variant of IbpB was incubated at 50 °C for 1 h. After that, the supernatant (S) and the pellet (P) were separated by centrifugation. The whole cell extract, supernatant, and pellet for each sample were then analyzed by Coomassie blue staining. (B) Semiquantification of relative chaperone‐like activity for the five Bpa variants of IbpB. The relative chaperone‐like activity was defined by the percentage of the soluble protein of the whole cell extract based on the Coomassie blue staining results from (A). Each experiment was repeated three times. Semiquantitative analysis data are presented as mean ± SEM.

To confirm that the insertion of Bpa at the selected residue positions does affect the known chaperone function of IbpB, we first determined the chaperone‐like activity of five Bpa variants of IbpB by measuring their abilities of suppressing heat‐induced aggregation of the whole cell extract prior to performing *in vivo* photo‐crosslinking. Wild‐type IbpB or its Bpa variants were overexpressed in *E. coli* BW25113‐Δ*ibpB* strain and cultured at 30 °C, and then, the whole cell extract was isolated and incubated at 50 °C for 1 h. After centrifugation, the soluble proteins and insoluble proteins were analyzed by Coomassie blue staining. As for Δ*ibpB* cells, only about half of the cell extract proteins remained soluble upon heat treatment (Fig. 2A, Lane 3), while almost all the cell extract proteins were kept in the soluble state when wild‐type IbpB was overexpressed (Fig. 2A, lane 6). This probably could be due to the high level of expression of wild‐type IbpB protein, which protected the cell extract proteins from aggregation, therefore unable to detect insoluble proteins in the pellet fraction. The protein level of Bpa variants in the cell extract varied with the Bpa incorporation site and is much lower than that of the wild‐type IbpB. However, most of the cell extract proteins were still maintained in the supernatant fraction (Fig. 2A, lanes 8, 11, 14, 17, 20). We further compared the relative chaperone‐like activity of the Bap variants by using semiquantitative analysis. Despite the relative low level of protein expression, all the Bpa variants exhibited over 75% chaperone‐like activities (Fig. 2B), indicating that the incorporation of Bpa has a negligible effect on the molecular chaperone function of IbpB.

### Substrate protein binding of IbpB in living cells is reversible upon short‐time exposure to 50 °C

**Fig. 3:**
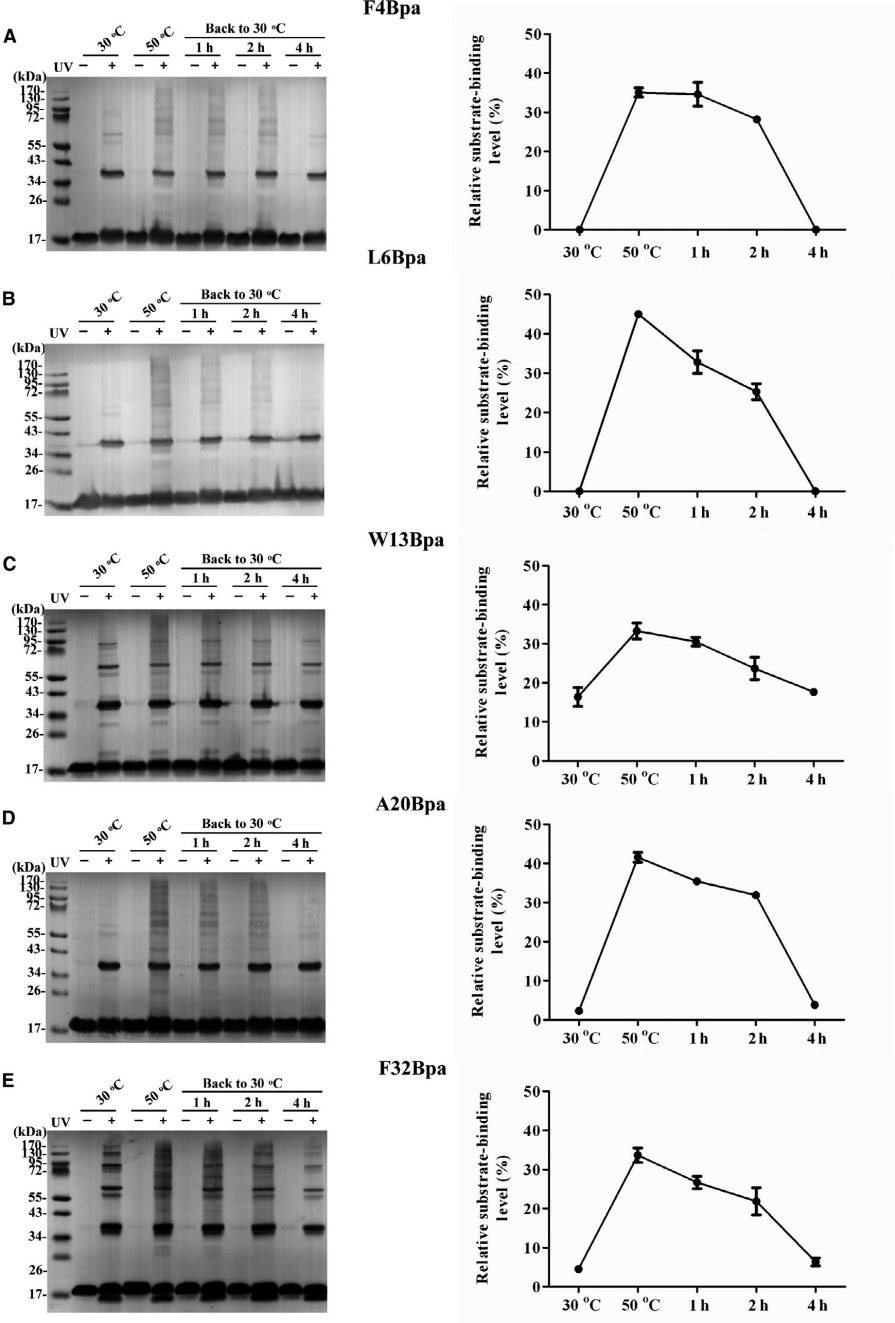
Characterization of the IbpB‐substrate interaction in living cells during recovery from heat shock. *Escherichia coli* Δ*ibpB* cells were grown at 30 °C with the presence of 0.02% arabinose to mid‐exponential phase, centrifuged at 2504 ***g*** for 1 min, resuspended and washed by fresh LB medium. The obtained cell cultures were exposed to heat shock at 50 °C for 10 min, cooled down on ice for 30 s and returned to the incubation at 30 °C. The cell cultures were sampled at different time points, i.e. before heat shock, right after heat shock and 1, 2, and 4 h after returning to 30 °C. All the samples were exposed to UV light irradiation for 10 min, resolved by SDS/PAGE and immunoblotted by anti‐His tag antibody. (A–E) The western blotting analysis of the crosslinking of F4Bpa, L6Bpa, W13Bpa, A20Bpa, and F32Bpa with cellular proteins and the semiquantification of relative substrate‐binding levels for each of the five Bpa variants of IbpB, respectively. Each experiment was repeated three times. Semiquantitative analysis data are presented as mean ± SEM.

Given that the chaperone‐like activity of IbpB has been shown to be maximal at 50 °C but almost undetectable at 30 °C [22], we selected 50 °C as the heat shock temperature and 30 °C as the recovery temperature. The *E. coli* cells expressing IbpB Bpa variants precultured at 30 °C were subjected to heat shock at 50 °C for 10 min and then transferred back to 30 °C for a prolonged incubation. The cell cultures were sampled at different time points (i.e., before heat shock, immediately after heat shock and 1, 2, 4 h after returning to 30 °C) and subsequently subjected to photo‐crosslinking analysis. We observed that after experiencing a heat shock treatment, the interaction of the tested residues Bpa with cellular proteins, as reflected by its photo‐crosslinked products (exception of crosslinked homo‐oligomers), was substantially decreased when returned to incubation for varying length of time at 30 °C, and this decrease in the *in vivo* photo‐crosslinked bound proteins showed different patterns, which is dependent on the locations of the residue. Specifically, the photo‐crosslinked products in the F4Bpa were not decreased during recovery for 1 and 2 hours until 4 hours (Fig. 3A). In contrast, the photo‐crosslinked products of other 4 Bpa variants were gradually decreased at indicated time upon a recovery from heat shock treatment (Fig. 3B–E). It could be seen that a 4‐h incubation at 30 °C enabled all these five IbpB Bpa variants to display almost the same crosslinking profile similar to that of the cells without heat shock. Besides, it should be mentioned that we have removed the arabinose after the induction of Bpa variants, avoiding the interference of newly synthesized variants in the crosslinking results during the prolonged incubation at 30 °C. Together, these data suggested that the substrate protein binding of IbpB in living cells was reversible upon short‐time exposure to 50 °C, which implied that the substrate proteins bound with IbpB upon heat shock stress and are released at nonstress conditions.

## Discussion

It has been found that IbpB was able to bind a wide spectrum of natural substrate proteins at 50 °C in living cells [23, 24]. Although it is believed that the substrate proteins which are bound to sHSPs would be released after the heat shock condition is removed, this interaction between sHSPs and their substrates during the recovery stage has rarely been examined *in vivo*. Here, by using Bpa‐mediated photo‐crosslinking, we characterize the interaction between IbpB and its endogenous substrates in living cells. Our data suggested that the substrate protein binding of IbpB *in vivo* was reversible upon short‐time exposure to 50 °C, providing the evidence that decreasing temperature leads IbpB to gradually hand off its bound aggregation‐prone and partially unfolded proteins. *In vitro* study demonstrated that IbpB‐bound proteins were stabilized in a conformation that can be subsequently released and specifically refolded by the DnaK‐DnaJ‐GrpE chaperones [15, 16]. However, it remains unknown how does the release of substrate take place *in vivo*. Further research needs to be done to reveal how the ATP‐dependent chaperones participate in this process.

IbpB, or sHSPs in general, function as a robust molecular chaperone to act upon a large diversity of substrate proteins in living cells growing under fluctuating conditions [23, 27], therefore making sHSP‐substrate interactions complex, and involving multiple sites on the sHSP. It has been demonstrated that the substrate‐binding residues of IbpB are located predominantly in the N‐terminal arm [23]. Here, we chose a total of five IbpB Bpa variants with Bpa incorporated in their N‐terminal arm, to investigate the IbpB‐substrate interaction. We found decrease in crosslinked cellular proteins in all tested IbpB Bpa variants when returned for further incubation at 30 °C after 50 °C heat stress. Fu et al. proposed according to their *in vivo* photo‐crosslinking data that there are three types of substrate‐binding residues in IbpB, classified into types I and II residues activated at low and normal temperatures, respectively, and type III residue mediated oligomerization at low temperature but switched to substrate binding at heat shock temperature [23]. In the N‐terminal arm of IbpB, all these three types of substrate‐binding residues are distributed. Here, we did not choose the type I residues, as they are capable of mediating substrate binding at 30 °C, that would make it not convenient to characterize the interaction dynamics of IbpB‐substrate from 30 °C to 50 °C, and back to 30 °C. In contrast, four type II residues (Phe‐4, Leu‐6, Trp‐13, and Ala‐20) and one type III residue (Phe‐32) which primarily mediate self‐oligomerization at 30 °C and switch to substrate binding at 50 °C were subjected to *in vivo* photo‐crosslinking analysis. Our results showed that, unlike F4Bpa, four other Bpa variants (L6Bpa, W13Bpa, A20Bpa, and F32Bpa) displayed the similar pattern in the decrease of photo‐crosslinked bound proteins during the recovery stage. That means the rate of substrate proteins release of these IbpB Bpa variants does not depend on the type of the residue with Bpa incorporated, suggesting that the interaction dynamics between IbpB and its substrates is complicated. We speculate that the positions of Bpa incorporation and the properties of bound substrates may be attributed to the difference in the substrate release rate. It seems that some substrates remain bound longer on certain residues of the IbpB N‐terminal arm. Since all the Bpa variants have been checked for function (as shown in Fig. 2), the complexity of the interaction between IbpB and its substrates is not due to the effect of Bpa incorporation.

Furthermore, this is the first study to investigate the substrate release of sHSPs in living cells by using photo‐crosslinking, which is an effective approach to obtain very dynamic and even short‐lived interactions that happen *in vivo*. Our study is of interest in methodology for investigating the interaction dynamics between molecular chaperones and their substrates in living cells.

## Conflict of interest

The authors declare no conflict of interest.

## Author contributions

XS and ANE designed the experiments. XS performed the *in vivo* photo‐crosslinking experiments. ANE performed the chaperone‐like activity assay. XS analyzed data. XS and ANE wrote the manuscript.

## Data Availability

Data supporting the findings of this manuscript are available from the corresponding authors upon reasonable request.
